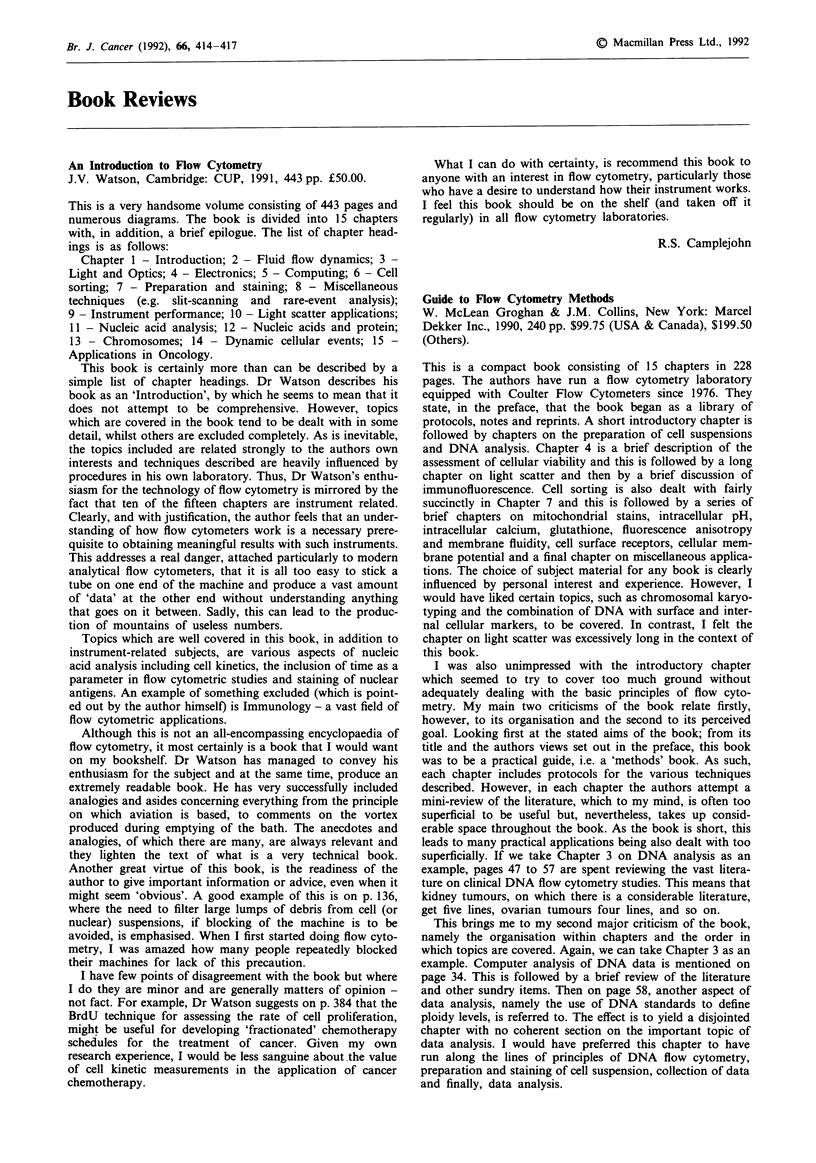# An Introduction to Flow Cytometry

**Published:** 1992-08

**Authors:** R.S. Camplejohn


 © Macmillan Press Ltd., 1992

### Book Reviews

**An Introduction to Flow Cytometry**
J.V. Watson, Cambridge: CUP, 1991, 443 pp. £50.00.

This is a very handsome volume consisting of 443 pages and numerous diagrams. The book is divided into 15 chapters with, in addition, a brief epilogue. The list of chapter headings is as follows:

Chapter 1 – Introduction; 2 – Fluid flow dynamics; 3 – Light and Optics; 4 – Electronics; 5 – Computing; 6 – Cell sorting; 7 – Preparation and staining; 8 – Miscellaneous techniques (e.g. slit-scanning and rare-event analysis); 9 – Instrument performance; 10 – Light scatter applications; 11 – Nucleic acid analysis; 12 – Nucleic acids and protein; 13 – Chromosomes; 14 – Dynamic cellular events; 15 – Applications in Oncology.

This book is certainly more than can be described by a simple list of chapter headings. Dr Watson describes his book as an 'Introduction', by which he seems to mean that it does not attempt to be comprehensive. However, topics which are covered in the book tend to be dealt with in some detail, whilst others are excluded completely. As is inevitable, the topics included are related strongly to the authors own interests and techniques described are heavily influenced by procedures in his own laboratory. Thus, Dr Watson's enthusiasm for the technology of flow cytometry is mirrored by the fact that ten of the fifteen chapters are instrument related. Clearly, and with justification, the author feels that an understanding of how flow cytometers work is a necessary prerequisite to obtaining meaningful results with such instruments. This addresses a real danger, attached particularly to modern analytical flow cytometers, that it is all too easy to stick a tube on one end of the machine and produce a vast amount of 'data' at the other end without understanding anything that goes on it between. Sadly, this can lead to the production of mountains of useless numbers.

Topics which are well covered in this book, in addition to instrument-related subjects, are various aspects of nucleic acid analysis including cell kinetics, the inclusion of time as a parameter in flow cytometric studies and staining of nuclear antigens. An example of something excluded (which is pointed out by the author himself) is Immunology – a vast field of flow cytometric applications.

Although this is not an all-encompassing encyclopaedia of flow cytometry, it most certainly is a book that I would want on my bookshelf. Dr Watson has managed to convey his enthusiasm for the subject and at the same time, produce an extremely readable book. He has very successfully included analogies and asides concerning everything from the principle on which aviation is based, to comments on the vortex produced during emptying of the bath. The anecdotes and analogies, of which there are many, are always relevant and they lighten the text of what is a very technical book. Another great virtue of this book, is the readiness of the author to give important information or advice, even when it might seem 'obvious'. A good example of this is on p. 136, where the need to filter large lumps of debris from cell (or nuclear) suspensions, if blocking of the machine is to be avoided, is emphasised. When I first started doing flow cytometry, I was amazed how many people repeatedly blocked their machines for lack of this precaution.

I have few points of disagreement with the book but where I do they are minor and are generally matters of opinion – not fact. For example, Dr Watson suggests on p. 384 that the BrdU technique for assessing the rate of cell proliferation, might be useful for developing 'fractionated' chemotherapy schedules for the treatment of cancer. Given my own research experience, I would be less sanguine about the value of cell kinetic measurements in the application of cancer chemotherapy.

What I can do with certainty, is recommend this book to anyone with an interest in flow cytometry, particularly those who have a desire to understand how their instrument works. I feel this book should be on the shelf (and taken off it regularly) in all flow cytometry laboratories.

R.S. Camplejohn